# Darinaparsin Is a Multivalent Chemotherapeutic Which Induces Incomplete Stress Response with Disruption of Microtubules and Shh Signaling

**DOI:** 10.1371/journal.pone.0027699

**Published:** 2011-11-15

**Authors:** Twila A. Mason, Elena Kolobova, Jiang Liu, Joseph T. Roland, Chin Chiang, James R. Goldenring

**Affiliations:** 1 Department of Cell and Developmental Biology, Vanderbilt University Medical Center, Nashville, Tennessee, United States of America; 2 Epithelial Biology Center, Vanderbilt University Medical Center, Nashville, Tennessee, United States of America; 3 Department of Surgery, Vanderbilt University Medical Center, Nashville, Tennessee, United States of America; The University of Kansas Medical Center, United States of America

## Abstract

Chemotherapeutics and other pharmaceuticals are common sources of cellular stress. Darinaparsin (ZIO-101) is a novel organic arsenical under evaluation as a cancer chemotherapeutic, but the drug's precise mechanism of action is unclear. Stress granule formation is an important cellular stress response, but the mechanisms of formation, maintenance, and dispersal of RNA-containing granules are not fully understood. During stress, small, diffuse granules initially form throughout the cytoplasm. These granules then coalesce near the nucleus into larger granules that disperse once the cellular stress is removed. Complete stress granule formation is dependent upon microtubules. Human cervical cancer (HeLa) cells, pre-treated with nocodazole for microtubule depolymerization, formed only small, diffuse stress granules upon sodium arsenite treatment. Darinaparsin, as a single agent, also induced the formation of small, diffuse stress granules, an effect similar to that of the combination of nocodazole with sodium arsenite. Darinaparsin inhibited the polymerization of microtubules both *in vivo* and *in vitro*. Interestingly, upon removal of darinaparsin, the small, diffuse stress granules completed formation with coalescence in the perinuclear region prior to disassembly. These results indicate that RNA stress granules must complete formation prior to disassembly, and completion of stress granule formation is dependent upon microtubules. Finally, treatment of cells with darinaparsin led to a reduction in Sonic hedgehog (Shh) stimulated activation of Gli1 and a loss of primary cilia. Therefore, darinaparsin represents a unique multivalent chemotherapeutic acting on stress induction, microtubule polymerization, and Shh signaling.

## Introduction

All cells experience various stressors during their physiological processes. Stress is a ubiquitous problem and can trigger a range of responses within cells. Common stressors include heat shock, oxidative conditions, viral infection, hypoxia, mitochondrial stress, DNA-damaging agents, and ultraviolet irradiation [1], [2]. Chemotherapeutic drugs and other pharmaceuticals are other common sources of cellular stress.

Arsenicals are one category of chemotherapeutics undergoing continual investigation. Organic and inorganic arsenicals have been used in the treatment of disease for thousands of years [3], [4]. However, arsenicals are typically very potent and their toxicity typically limits their use [4], [5]. Currently, arsenic trioxide (ATO, As_2_O_3_), an inorganic arsenical, is used for treatment in cases of recurrent acute promyelocytic leukemia [6]. This drug is limited by its toxicity on the liver and cardiovascular system. Because of these toxicity issues, the development of less toxic arsenicals has become an area of great interest.

Darinaparsin (ZIO-101, S-dimethylarsino-glutathione) is an organic arsenical that is currently in Phase II clinical trials as a chemotherapeutic agent [5], [7]. While darinaparsin shows clinical promise, the drug's mechanism of action continues to be investigated. A series of in vitro and in vivo studies, coupled with observed differences in toxicity and potency, suggest darinaparsin employs a mechanism of action that is different from that of ATO. Darinaparsin appears more potent than ATO as some ATO-resistant cells are sensitive to the drug. Moreover, other cell types have shown an increased sensitivity to darinaparsin compared to ATO. [5], [6], [8], [9]. Importantly darinaparsin does not demonstrate the same level or type of toxicity as ATO with no apparent toxicity to the liver or cardiovascular system.

Previous studies have shown that both ATO and darinaparsin cause apoptosis and cell cycle arrest at G_2_/M in tumor cells [4], [6]. This effect is thought to occur in part through the disruption of mitochondrial functions, which leads to increased production of reactive oxygen species (ROS) [10]. An increase in ROS, if great enough, can trigger a cellular stress response. Recent preclinical studies of darinaparsin have shown anti-angiogenic activity in vitro and in vivo through controlling several signal transduction pathways [6] and that darinaparsin is more potent than ATO under hypoxic conditions [11].

One critical mechanism in the cellular stress response is the formation of RNA stress granules. Stress granules function to sequester mRNAs and proteins during cellular stress. Importantly, stress granules are not stable structures, but are dynamic with a constant flux of proteins and mRNAs. Stress granules are generally composed of a variety of preinitiation- and translation-related factors, proteins linked to mRNA metabolism, mRNAs, and RNA-binding proteins, as well as signaling proteins with no known link to mRNA [2]. Typically, proteins and mRNAs necessary for cell survival during stress, such as heat shock proteins, are not sequestered in stress granules. The mRNAs are sequestered in stress granules until the cell is no longer stressed or undergoes apoptosis [12]. Once stress is no longer present, stress granules disperse, and the sequestered mRNAs can be translated. Other RNA granules also exist in cells without stress, such as processing bodies (P-bodies) [1]. The different RNA granules do contain unique components, with stress granules containing components specialized for adaptation to dynamic stressors. Though stress granule formation appears to be a ubiquitous cellular response, the mechanisms regulating formation, maintenance, and dispersal remain largely obscure.

In this study, we examined both the mechanism of action for darinaparsin and the dynamics of stress granules formation, maintenance, and dispersal. We propose that darinaparsin is a potent chemotherapeutic because it initiates a stress response, while also stalling the completion of the stress response by simultaneously depolymerizing microtubules. The depolymerization of microtubules causes the stress granules to form incompletely, preventing the coalescence of stress granule components. When darinaparsin is withdrawn, stress granules progress to final coalescence as microtubules repolymerize and disperse only after complete granule formation. In addition, likely due to microtubule depolymerization, darinaparsin causes a loss of primary cilia and a reduction in Shh signaling. These findings support the concept that darinaparsin is a multivalent chemotherapeutic affecting several critical processes in neoplastic cells.

## Results

### Darinaparsin and sodium arsenite induce stress granule formation

**Figure 1:**
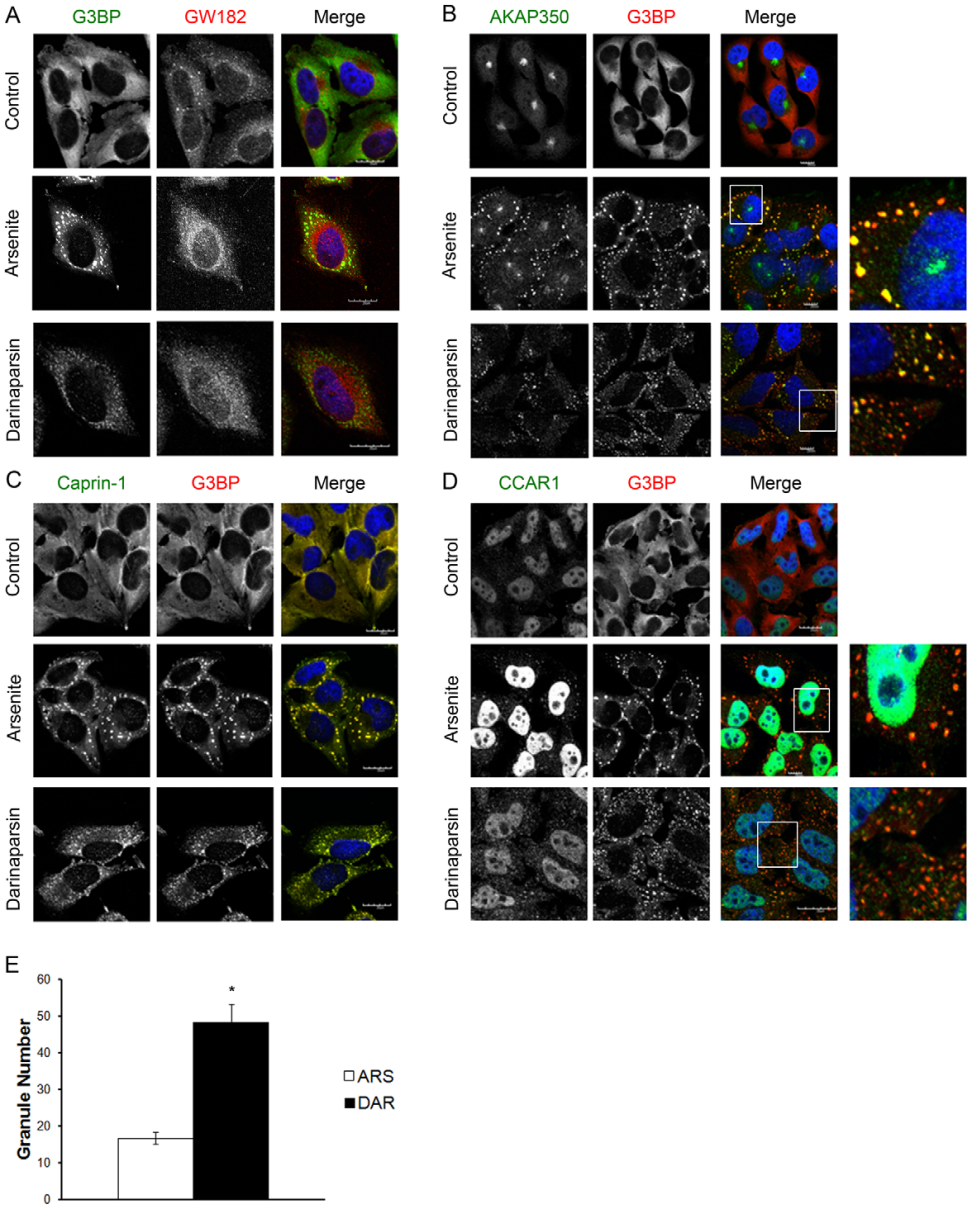
Darinaparsin and sodium arsenite induce RNA stress granules. HeLa cells were treated with 0.5 mM Sodium arsenite or 0.05 mM darinaparsin for 45 minutes at 37°C. Cells were fixed with supplemented 4% PFA. All cells are stained for DNA with Dapi (blue) and **A.** G3BP (green) and GW-182 (red), **B.** AKAP350 (green) and G3BP (red), **C.** Caprin-1 (green) and G3BP (red), **D.** CCAR1 (green) and G3BP (red). **E.** The number of granules in sodium arsenite- and darinaparsin-treated cells was quantified using ImageJ software. Results are mean +/− standard error. *p<0.0001 (Scale bars: 20 µM).

Since darinaparsin is an organic arsenical, we first sought to evaluate whether darinaparsin could elicit RNA stress granules. To determine the effect of darinaparsin on cells, we treated cells with darinaparsin and compared its effects to those of sodium arsenite. Treatment of HeLa cells with 0.05 mM darinaparsin for 30 minutes induced stress granule formation, detected with antibodies against G3BP (Figure 1). A titration of darinaparsin for short incubations showed stress granule formation was induced in a majority of cells at 0.03 mM. Sodium arsenite treatment at 0.5 mM induced stress granule formation, as previously reported [1], [2]. Nevertheless, the stress granules induced by darinaparsin appeared smaller and were significantly greater in number when compared to sodium arsenite-induced stress granules, and the granules were more dispersed throughout the cytoplasm (Figure 1E).

As noted above, stress granule formation is a dynamic process. Upon initial stress granule induction, many small granules form throughout the cytoplasm. As stress continues, the smaller granules coalesce near the nucleus to form large, complete stress granules [1]. The large, complete stress granules generally have similar compositions, but the smaller granules can vary in composition [1]. Because stress granules can vary in composition, we evaluated the stress granules induced by darinaparsin in HeLa cells compared with stress granules induced by sodium arsenite, and cells that received no treatment served as a control (Figure 1). We first studied the association of markers of Processing bodies (P bodies) with induced stress granules. P-bodies serve as RNA degradation centers and are present in the absence or presence of cellular stress [2], [13]. Stress granules and P-bodies are separate entities, but they do communicate with one another and are dynamically linked [14]. To ensure the granules induced by darinaparsin were not P-bodies, we compared staining of a P-body marker, GW182, and a stress granule marker, G3BP (Figure 1A). In untreated cells, GW182-stained P-bodies were small and distributed throughout the cytoplasm, while G3BP was diffuse throughout the cytosol. Sodium arsenite treatment induced large G3BP-staining stress granules that clustered in the perinuclear region. Only a few of the larger stress granules showed dual immunostaining with both G3BP and GW182. Similarly, there was little overlap between the smaller darinaparsin-induced granules and GW182-labelled P-bodies.

We next examined the presence of proteins previously found in stress granules in the granules induced by darinaparsin. AKAP350A, a large scaffolding protein, localizes to stress granules and is important for their complete formation [1]. Knockdown of AKAP350A with siRNA results in smaller stress granules upon treatment with sodium arsenite. AKAP350A colocalized with G3BP in stress granules induced by sodium arsenite (Figure 1B). After darinaparsin treatment, some granules contained both AKAP350A and G3BP, while others contained only one of the components. Granules containing only one component were typically smaller and in the periphery of the cytoplasm. AKAP350A interacts with RNA-binding proteins Caprin-1 and CCAR1, both of which are also present in stress granules [1], [15], [16]. Caprin-1 colocalized with G3BP in stress granules induced by both sodium arsenite and darinaparsin (Figure 1C). CCAR1 and G3BP colocalized in stress granules induced by sodium arsenite (Figure 1D). However, as with AKAP350A, in darinaparsin-treated cells some stress granules contain both CCAR1 and G3BP, but many smaller granules stained for only one of the components.

### Darinaparsin inhibits microtubule polymerization

**Figure 2:**
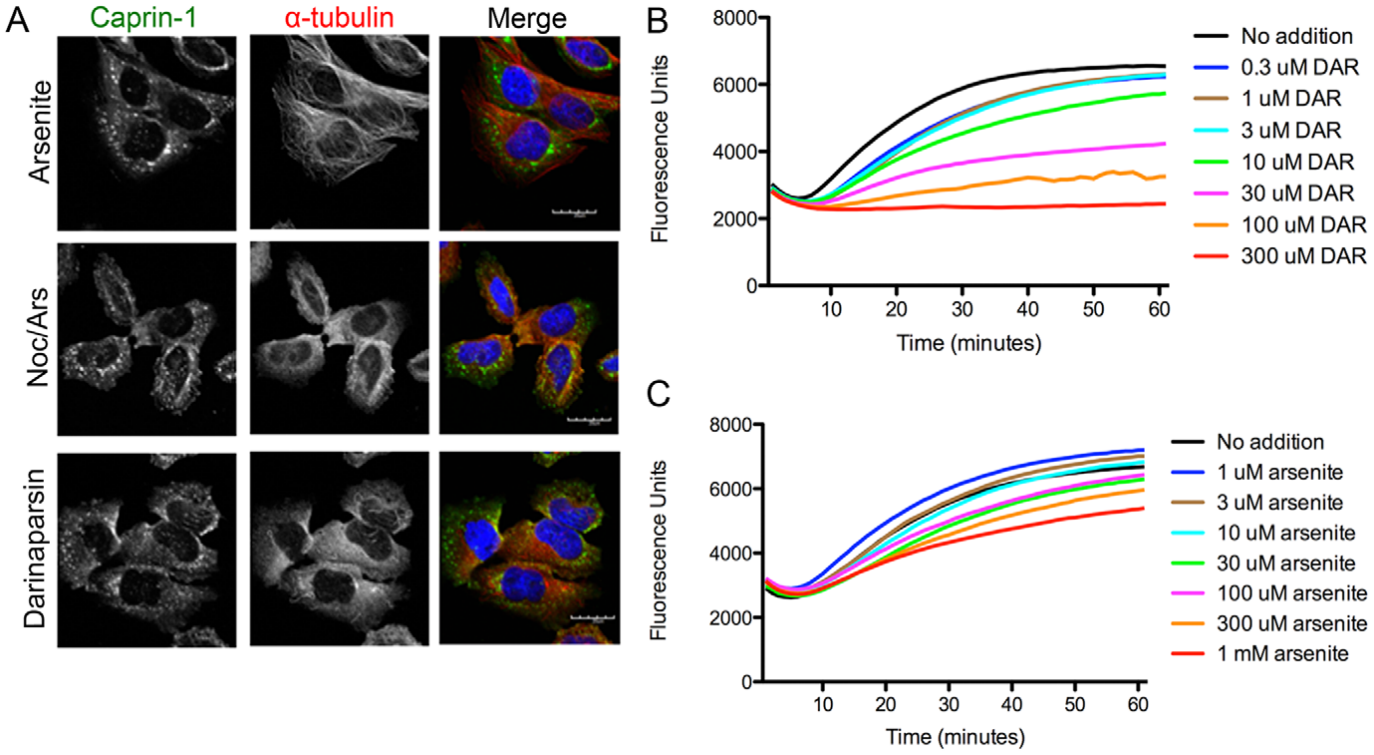
Darinaparsin inhibits microtubule polymerization. **A.** HeLa cells were treated with 0.5 mM Sodium arsenite or 0.05 mM darinaparsin, or 33 nM Nocodazole/0.5 mM Sodium arsenite. Cells are stained for DNA (Dapi, blue), a-tubulin (red), and Caprin-1 (green; stress granule marker). GTP-initiation microtubule polymerization in the presence of darinaparsin (**B**) and sodium arsenite (**C**) was measured *in vitro*. (Scale bars: 20 µM).

In previous studies, we have shown that microtubules are necessary for complete stress granule formation [1]. Pre-treatment of cells with nocodazole, which inhibits microtubule polymerization, prior to treatment with sodium arsenite, leads to the formation of stress granules which are smaller and greater in number. The smaller granules suggest incomplete stress granule formation. The similarity between darinaparsin-induced and nocodazole/sodium arsenite-induced stress granules led us to hypothesize that darinaparsin induces incomplete stress granule formation due to microtubule depolymerization. Immunofluorescence in HeLa cells demonstrated that treatment with darinaparsin did induce microtubule depolymerization along with inducing stress granule formation (Figure 2A). In cells treated with darinaparsin, microtubules were almost completely depolymerized, showing mainly cytosolic alpha-tubulin staining. In contrast, sodium arsenite treatment had no demonstrable effect on microtubules.

To verify the effects of darinaparsin on microtubules, we assessed *in vitro* microtubule polymerization (Figure 2B). Darinaparsin inhibited GTP-stimulated microtubule polymerization in a concentration-dependent manner. Darinaparsin also inhibited polymerization of microtubules stimulated by taxol, a microtubule stabilizer, at similar concentrations (data not shown). These results suggest that darinaparsin sequesters free tubulin monomers, similar to the action of nocodazole [17]. In contrast, sodium arsenite had only minimal effect on microtubule polymerization at concentrations up to 1 mM. Importantly, darinaparsin had no ability *in vitro* to depolymerize already polymerized microtubules (data not shown).

### Stress granules must complete formation before dispersal

We have previously shown that stress granules initially form as small granules in the cytosol, that then coalesce into the final, complete stress granules in the perinuclear region [1]. We now sought to evaluate stress granule dynamics further by investigating their dispersal once stress is relieved. Because darinaparsin, sodium arsenite, and nocodazole/sodium arsenite have differing effects, we monitored the dynamics of stress granule formation and dispersal during each treatment.

**Figure 3:**
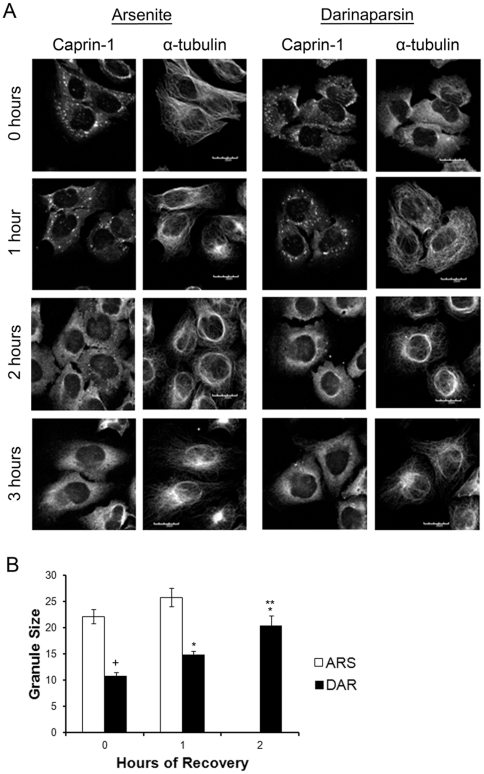
Stress granules must complete formation before dispersal. HeLa cells were treated with 0.5 mM Sodium arsenite or 0.05 mM darinaparsin. The 0 hour timepoint was taken at the end of treatment. For recovery, cells were incubated in complete RPMI media. Cells were fixed at timepoints with supplemented 4% PFA and stained for Caprin-1 and α-tubulin (**A**). **B.** Granule size was quantified for each hour of recovery using ImageJ software. There were no granules at 2 hours of recovery from sodium arsenite. Results are mean +/− standard error. +p<0.0001 vs. sodium arsenite-treated. *p<0.0001 vs. darinaparsin 0 hours of recovery. **p<0.0001 vs. darinaparsin 1 hour of recovery. (Scale bars: 20 µM).

We assessed the course of recovery of HeLa cells after treatment with 0.05 mM darinaparsin, 0.5 mM sodium arsenite, or a combination of 33 nM nocodazole and 0.5 mM sodium arsenite. Cells were fixed at the end of treatment for 45 minutes and after periods of recovery without treatment for up to three hours in complete media (Figure 3). At the end of treatment, sodium arsenite-treated cells had large, complete stress granules and intact microtubules, as expected (Figure 3). After recovering without drug for one hour, the sodium arsenite-treated cells showed fewer stress granules, and by two hours of recovery, the stress granules had completely dispersed into the cytoplasm. The microtubules remained unaffected by the sodium arsenite treatment for the duration of the experiment.

In cells treated with darinaparsin, the microtubules were depolymerized and stress granules were significantly smaller than sodium arsenite-induced stress granules at the end of treatment (Figure 3). After one hour of recovery in the absence of drug, the microtubules in cells treated with darinaparsin were repolymerized, and larger stress granules were now observed, especially in the perinuclear region. After three hours of recovery, darinaparsin treated cells fully recovered with dispersal of stress granules. Similar results were obtained in recovery from the combination of treatment with both nocodazole and sodium arsenite, but recovery occurred more slowly (data not shown). The dynamics of stress granule formation are more clearly seen in continuous live cell imaging. Movie S1 demonstrates that, following removal of darinaparsin, small G3BP-labeled stress granules first coalesced in the perinuclear region and then dispersed. These results suggest that stress granules must complete formation by coalescence along microtubule-dependent tracks before they can disperse into the cytoplasm.

### Darinaparsin affects Sonic Hedgehog signaling

**Figure 4:**
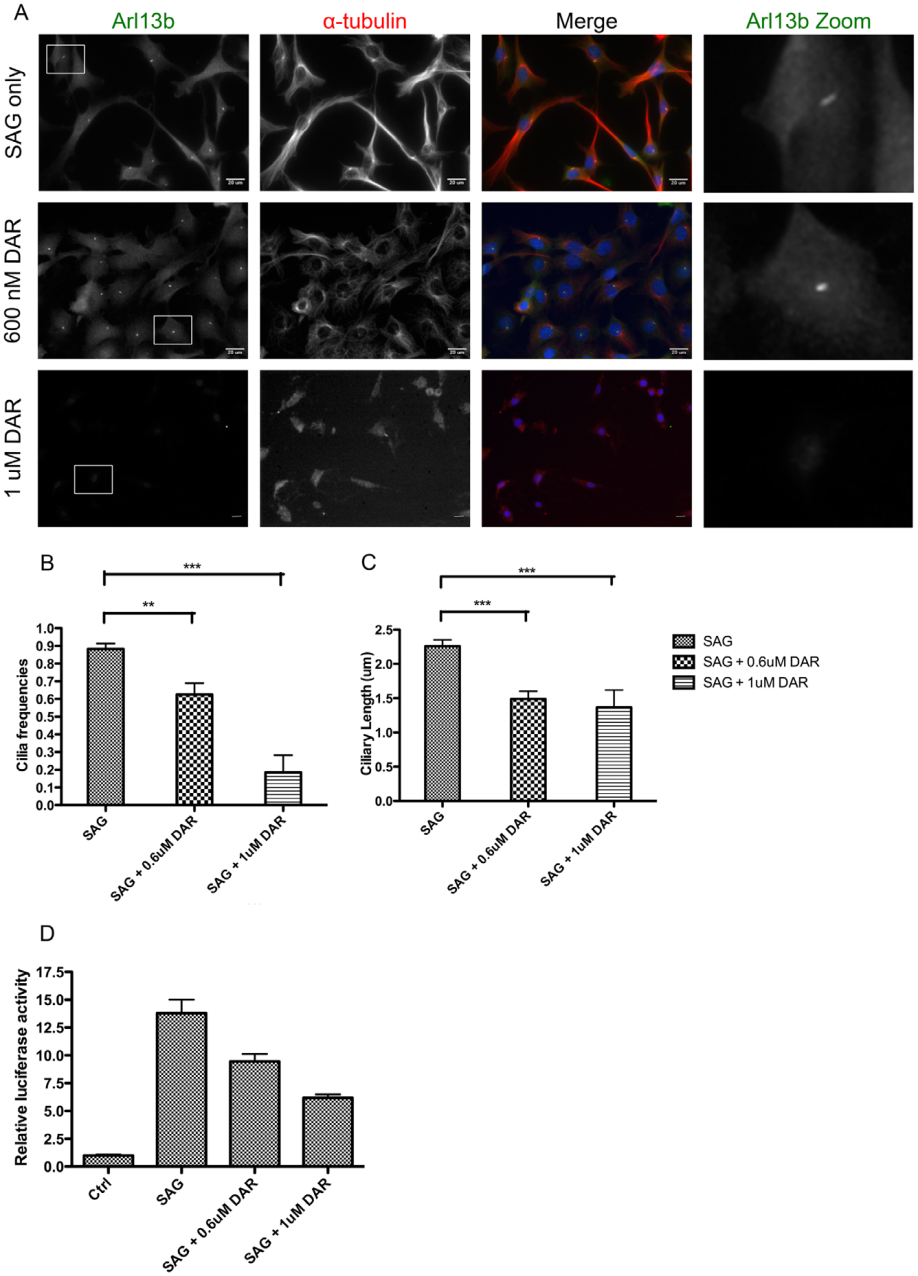
Darinaparsin inhibits Sonic Hedgehog signaling and primary cilia formation. NIH 3T3 fibroblasts were treated with 1001 nM SAG (Shh agonist) with or without 0.6 µM or 1 µM darinaparsin (ZIO-101). **A.** Cells were fixed with 4% PFA and stained for DNA (Dapi; blue), primary cilia (Arl13b; green), and α-tubulin (red). **B.** The number of primary cilia was determined from 6 samples per genotype (15–30 cells per sample). **C.** The length of primary cilia was determined using Image J software. P values were calculated using unpaired t-test with n–1 degree of freedom (GraphPad). **p<0.005, ***p<0.0001. **D.** Luciferase assays were performed in the absence or presence of darinaparsin. (Scale bars: 20 µM).

Recent investigations have reported that ATO inhibits hedgehog signaling [18], [19]. We sought to investigate whether darinaparsin also affects hedgehog signaling. Previous studies have shown that ATO does not affect primary cilium formation in mouse embryonic fibroblasts (MEFs) [18]. To test the effects of darinaparsin on primary cilia, NIH 3T3 cells were grown and induced to form primary cilia. The cells were treated with 100 nM Smoothened Agonist (SAG) with or without 600 nM or 1 µM darinaparsin for 24 hours [20]. The concentration of darinaparsin needed for long incubations was titrated, and 600 nM was the optimal concentration to have an effect while not causing cell death. Fixed cells were stained for Arl13b to mark primary cilia. Unlike cells treated with ATO, cells treated with 600 nM darinaparsin had a significant decrease in the number of primary cilia. There was further loss of primary cilia at 1 µM darinaparsin, suggesting a concentration-dependent decrease in primary cilia (Figure 4). In darinaparsin-treated cells that did form primary cilia, the primary cilia were shorter in length. Similar to ATO [18], darinaparsin treatment did not alter the intracellular localization of Gli1 (data not shown). Given the loss of primary cilia observed with darinaparsin treatment, we also evaluated the drug's effect on Shh signaling through an assay of Gli1 activation. Figure 4 demonstrates that darinaparsin elicited a concentration-dependent inhibition of Shh signaling, as seen with ATO [19]. These results indicate that darinaparsin has broad effects on stress granule formation, microtubule dynamics and Shh signaling.

### Darinaparsin effects are independent of Mdr1 expression

**Figure 5:**
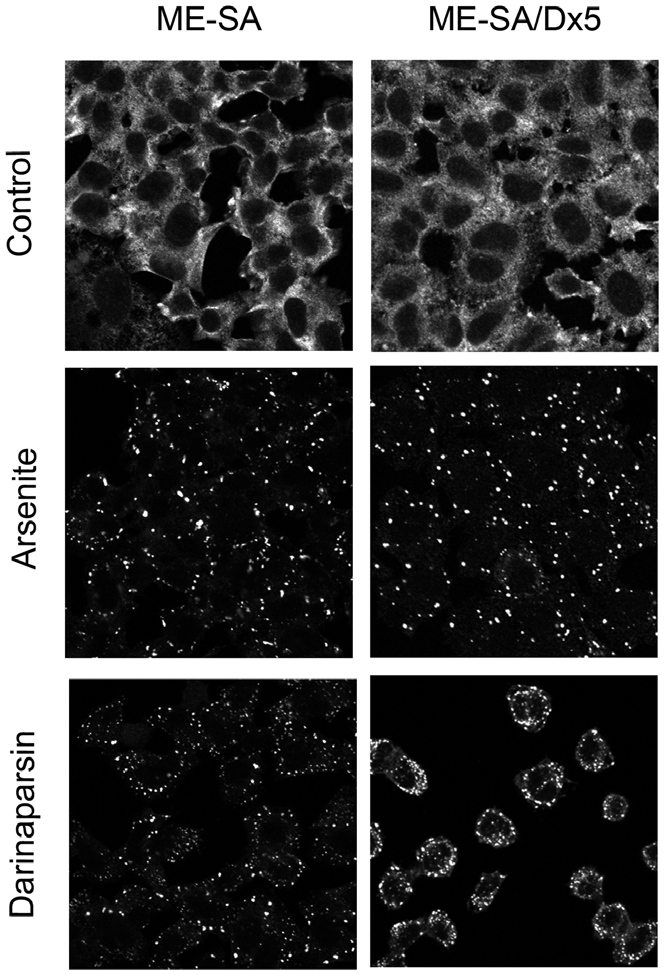
Darinaparsin is effective in Mdr-1-expressing cells. ME-SA and ME-SA/Dx5 cells were untreated or treated with 0.5 mM Sodium arsenite or 0.05 mM darinaparsin for 30 minutes at 37°C. Cells were fixed with supplemented 4% PFA and stained for G3BP.

Darinaparsin is a multivalent drug that appears to be more potent than similar drugs, such as ATO. Therefore, we tested its efficacy in cells with multiple drug resistance. Multiple drug resistance transporters are often a limiting factor on the practical efficacy of chemotherapeutic agents, especially in recurrent cancers [21]. While ATO is used to treat hematological malignancies, its use is limited in cancers expressing the multiple drug resistance protein, MRP-1 [8]. MRP1-expressing cells are resistant to ATO treatment, but are sensitive to treatment with darinaparsin [8]. This prompted us to examine another major drug resistance protein, Mdr1, which can rapidly pump drugs out of resistant cancer cells. Previous investigations have shown that ATO is not a substrate for Mdr1, and thus is effective in Mdr1-expressing cells [21]. We evaluated the effects of darinaparsin on stress granule formation in uterine sarcoma cells lines with or without expression of Mdr1. Figure 5 demonstrates that darinaparsin and sodium arsenite induced stress granules in both ME-SA cells, which do not express Mdr1, and ME-SA/Dx5 cells, which strongly over-express Mdr1. Indeed, the Mdr1-over-expressing cells appeared to be more susceptible to darinaparsin treatment as shown by the rapid rounding up of these cells after treatment. Along with previous studies, these results demonstrate that darinaparsin effects are not altered by expression of multiple-drug resistance transporters.

## Discussion

Arsenic salts are one of the oldest pharmaceutical classes [3]. Treatment with arsenicals has been utilized for both infectious diseases and cancer. While ATO is approved for treatment of refractory leukemias, its toxicity limits its use [6]. In contrast, darinaparsin, an organic arsenical, shows a wide range of potency against many cancer cells and has low toxicity when administered in humans [6]. While darinaparsin induces apoptosis and G_2_/M arrest in a number of cell lines, the exact mechanism responsible for the anti-neoplastic effects of the compound seem to vary according to cell types [6]. The present investigation indicates that darinaparsin acts as a multivalent pharmaceutical that induces a stress response, but does not permit its completion due to its influence as a microtubule polymerization inhibitor. Microtubule depolymerization by darinaparsin also causes loss of primary cilia and Shh signaling, likely due to a loss of Smoothened presentation. Inhibition of microtubule polymerization by darinaparsin likely accounts for the arrest of cells in G_2_/M noted in previous studies [6]. The G_2_/M arrest is characteristic of microtubule depolymerization inhibitors such as nocodazole [22]. Together these effects make this organic arsenical drug a potent chemotherapeutic with multiple mechanisms that target key regulators of neoplasia.

Formation of RNA stress granules following various cellular stressors is considered a critical adaptive mechanism in cell survival. Stress granules accumulate translational intermediates including critical mRNAs that are important for reestablishment of cell function after the release of stress [2]. Darinaparsin induces stress granule formation and simultaneously inhibits microtubule polymerization. This scenario leads to incomplete formation of stress granules. The inability to form stress granules or incomplete stress granule formation likely leads to apoptosis in affected cells [12]. Importantly, the unique property of darinaparsin to stimulate incomplete stress granule formation has allowed us to investigate the process of stress granule processing after removal of the blockade. Removal of darinaparsin led to completion of stress granule formation, with coalescence of smaller granules into larger perinuclear granules, coincident with the repolymerization of microtubules. These results are consistent with our previous results that stress granules form initially in the cell periphery and then coalesce after centripetal transport along microtubule tracks [1]. Following withdrawal of darinaparsin, stress granules only dissipated after completion of granule formation. These findings indicate that stress granules must complete their full program of formation and coalescence prior to subsequent disassembly.

Recent investigations have noted that arsenic trioxide can inhibit Shh signaling [18], [19]. Similarly, we have observed that darinaparsin also strongly inhibits Shh-induced Gli1 expression. Nevertheless, in contrast with arsenic trioxide, darinaparsin also causes the loss of microtubules and the primary cilium, the site of Smoothened presentation [18], [19]. Since Shh is considered a major driver in multiple cancers, it appears that darinaparsin has the ability to dismantle key aspects of Shh signaling machinery necessary for the proliferative drive in neoplastic cells.

Darinaparsin appears to be more potent but less toxic than ATO. Previous studies have noted that darinaparsin and ATO both induce an increase in ROS, but darinaparsin produces a greater increase [4]. Darinaparsin is a potent inhibitor of microtubule polymerization while the effects of ATO on microtubules can vary. Some studies have reported that ATO is a strong microtubule polymerizing agent that synergizes with taxol, while others have shown ATO inhibition of the effects of taxol [23], [24], [25]. Also, darinaparsin is effective in cells with ATO-resistance [8], [9]. Part of this latter difference may be due to the ability of some multiple drug resistance transporters, such as MRP1, to transport ATO out of cells [9]. In contrast, the expression of MRP1 does not alter the effects or potency of darinaparsin [8]. As noted previously for ATO [21], effectiveness of darinaparsin is not altered in Mdr1-expressing cells. In clinical trials, darinaparsin appears less toxic than ATO [6]. In general, organic arsenicals, such as darinaparsin, are typically less toxic than inorganic arsenicals, such as ATO [9]. The lower toxicity of darinaparsin may seem a bit counterintuitive given its broad range of activities, including strong action against microtubule polymerization. Indeed, the differences in practical therapeutic effects of darinaparsin may accrue from a relative therapeutic index of multiple specific target activities impacting neoplastic cells versus non-specific generalized effects of arsenicals affecting mitochondrial functions [6]. In any case, the range of actions of darinaparsin against multiple drivers of neoplastic cells may make this organic arsenical an effective therapy for multiple resistant solid and hematological malignancies.

## Methods

### Cell Culture

HeLa cells (American Type Culture Collection, ATCC) were maintained at 37°C in 5% CO_2_ using complete RPMI media supplemented with 10% fetal bovine serum (FBS). Polyjet was used for transfections according to the manufacturer's protocol (SignaGen). NIH 3T3 fibroblasts were maintained in high-glucose Dulbecco's modified Eagle's (DMEM) medium supplemented with 10% FBS. To induce cilia formation, 3T3 cells were grown on poly-*L*-lysine coated coverslips and maintained in DMEM medium supplemented with 0.5% calf serum. ME-SA and ME-SA/Dx5 cells (American Type Culture Collection, ATCC) were maintained at 37°C in 5% CO_2_ using complete McCoy's media supplemented with 10% FBS.

### Cell stress and recovery

For stress induction, HeLa cells were incubated at 37°C for 45 minutes (fixed cells) or 30 minutes (live cells) with a final concentration of either 0.5 mM sodium arsenite (Riedel de Haen) or 0.05 mM darinaparsin (Ziopharm). For microtubule depolymerization, cells were first incubated on ice for 30 minutes. Then, cells were treated with 33 nM nocodazole (Calbiochem) for 90 minutes at 37°C. In recovery experiments, cells were washed twice with serum-free media after treatment, and incubated in complete RPMI media at 37°C for duration of recovery. For primary cilia staining, 3T3 cells were treated with100 nM Smoothened Agonist (SAG) [20], and with or without darinaparsin 18 hours before staining.

### Fluorescence microscopy and analysis

For stress and recovery studies, HeLa cells grown on coverslips were fixed at room temperature for 15 minutes using 4% paraformaldehyde (PFA) supplemented with 0.1% Triton X-100, 80 mM K-PIPES pH 7.2, 1 mM EGTA, 1 mM MgSO_4_, and 30% glycerol. Cells were permeabilized with 0.25% Triton X-100 and blocked with 5% normal serum for one hour at room temperature. The cells were incubated with primary antibodies for one hour at room temperature: rabbit anti-AKAP350A (1∶200), rabbit anti-Caprin-1 (1∶1000), mouse anti-α-tubulin (1∶5000, Sigma), rabbit anti-CCAR1 (1∶1000), goat anti-GW182 (1∶100, Santa Cruz), and mouse anti-G3BP (1∶800, BD Transduction). This was followed by incubation at room temperature for one hour with species-specific fluorescent secondary antibodies (1∶500; Invitrogen or Jackson Immunoresearch). Coverslips were mounted using Prolong Gold with DAPI (Invitrogen). Cells were imaged using a 60× oil immersion lens on Olympus FV-1000 confocal fluorescence microscope (Vanderbilt Cell Imaging Shared Resource). The number and average size of granules in sodium arsenite- and darinaparsin-treated cells were determined using ImageJ software (NIH). At least ten cells were used per calculation. Changes in granule number and size were analyzed using a Student's t-test. Changes in granule size over time in darinaparsin-treated cells were analyzed using a one-way analysis of variance (ANOVA) with post hoc examination of significant means using Tukey's test. Live-cell imaging was performed using a 60× oil immersion lens on the DeltaVision Deconvolution microscope (Vanderbilt Epithelial Biology Center Imaging Resource).

For primary cilia staining, 3T3 cells grown on coverslips were fixed for 20 minutes in 4% paraformaldehyde and permeabilized for 15 minutes in PBS with 0.3% Triton-X 100. The cells were incubated in primary antibodies overnight at 4°C: rabbit-anti-Arl13b (1∶2000; gift from Tamara Caspary, Emery University) and mouse-anti-α-tubulin (1∶5000; Calbiochem). This was followed by incubation at room temperature for one hour with species-specific fluorescent secondary antibodies (1∶1000; Invitrogen). DAPI was included in the final washes before the samples were mounted in Fluor Saver (Calbiochem) for microscopy. Images were taken with Opti-Grid Confocal microscope. Cilia frequencies were determined from Arl13b/DAPI- stained cells from 6 samples per treatment (15–30 cells per sample). Ciliary length was measured using NIH ImageJ software. Bases and ends of immunolabeled individual cilia were marked in the confocal z-stack images and outlined, and the calculated length was recorded. P values were calculated using unpaired t-test (GraphPad).

### Microtubule polymerization

The effects of darinaparsin on microtubule polymerization were assayed using a fluorescence-based polymerization assay (Cytoskeleton, Inc). Microtubule polymerization was initiated by GTP or GTP/Taxol (1 µM) in the presence of increasing concentrations of either darinaparsin (0.3 µM to 300 µM) or arsenite (1 µM to 1 mM). Polymerization was monitored each minute by fluorescence measurement for a total of one hour in a Biotek Fluorescence plate reader.

### Luciferase reporter assay

NIH3T3 cells were plated at 1.0×10^5^ cells/ml (24-well plate) 16 hours before transfection. Cells were co-transfected with 0.3 µg 8XGli1-luciferase reporter construct and 0.03 µg Renilla-luciferase control construct in each well. At 8 hours after transfection, cells were fed low serum medium (0.5% calf serum + DMEM), and then treated with Shh agonist (10 nM SAG) with or without darinaparsin for an additional 20 hours. Luciferase assays were performed with the Dual-Glo Luciferase System (Promega) according to the manufacturer's instructions. Each assay was performed in triplicate and the results are presented as the relative fold-increase (normalized to Renilla-luciferase activity) over non-SAG-treated cells.

## Supporting Information

Movie S1 Stress granules must complete formation before dispersal. HeLa cells were transfected with pcherry-G3BP using Effectene. Cells were treated with 0.05 mM darinaparsin for 30 minutes, washed with serum-free media twice, then incubated in complete media for recovery.
(MOV)
